# Intralymphatic GAD-alum Injection Modulates B Cell Response and Induces Follicular Helper T Cells and PD-1+ CD8+ T Cells in Patients With Recent-Onset Type 1 Diabetes

**DOI:** 10.3389/fimmu.2021.797172

**Published:** 2022-01-12

**Authors:** Hugo Barcenilla, Mikael Pihl, Jeanette Wahlberg, Johnny Ludvigsson, Rosaura Casas

**Affiliations:** ^1^ Division of Pediatrics, Department of Biomedical and Clinical Sciences, Faculty of Medicine and Health Sciences, Linköping University, Linköping, Sweden; ^2^ Core Facility, Flow Cytometry Unit, Linköping University, Linköping, Sweden; ^3^ Department of Health, Medicine and Caring Sciences (HMV), Linköping University, Linköping, Sweden; ^4^ Division of Diagnostics and Specialist Medicine and Faculty of Health Sciences, Örebro University, Örebro, Sweden; ^5^ Division of Pediatrics, Crown Princess Victoria Children’s Hospital, Linköping, Sweden

**Keywords:** antigen-specific immunotherapy, type 1 diabetes, GAD-alum, mass cytometry (CyTOF), follicular T helper cells, T cell exhaustion, B cell response, T1D

## Abstract

Antigen-specific immunotherapy is an appealing strategy to preserve beta-cell function in type 1 diabetes, although the approach has yet to meet its therapeutic endpoint. Direct administration of autoantigen into lymph nodes has emerged as an alternative administration route that can improve the efficacy of the treatment. In the first open-label clinical trial in humans, injection of aluminum-formulated glutamic acid decarboxylase (GAD-alum) into an inguinal lymph node led to the promising preservation of C-peptide in patients with recent-onset type 1 diabetes. The treatment induced a distinct immunomodulatory effect, but the response at the cell level has not been fully characterized. Here we used mass cytometry to profile the immune landscape in peripheral blood mononuclear cells from 12 participants of the study before and after 15 months of treatment. The immunomodulatory effect of the therapy included reduction of naïve and unswitched memory B cells, increase in follicular helper T cells and expansion of PD-1+ CD69+ cells in both CD8+ and double negative T cells. *In vitro* stimulation with GAD_65_ only affected effector CD8+ T cells in samples collected before the treatment. However, the recall response to antigen after 15 months included induction of CXCR3+ and CD11c+Tbet+ B cells, PD-1+ follicular helper T cells and exhausted-like CD8+ T cells. This study provides a deeper insight into the immunological changes associated with GAD-alum administration directly into the lymph nodes.

## Introduction

Type 1 diabetes (T1D) is a chronic autoimmune disease that causes severe complications despite intensive treatment. Antigen-specific immunotherapy is a safe and well-tolerated therapeutic strategy to prevent beta-cell loss (1, 2). Subcutaneous administration of glutamic acid decarboxylase (GAD)65 formulated with aluminum hydroxide (GAD-alum) has been evaluated in several clinical trials, although with varied results (3–5). However, a meta-analysis of published results from these trials did not rule out the therapeutic benefit of GAD-alum therapy (6). The need to improve the efficacy of the therapy led to the first in-human open-label clinical trial in which the autoantigen was delivered directly into a lymph node (DIAGNODE-1) in individuals with recent onset T1D. The treatment was safe and tolerable, and resulted in promising preservation of C-peptide after 15 months (7).

Compared to subcutaneous administration, intralymphatic delivery of GAD-alum has enhanced immunogenicity, requiring lower doses of GAD to induce antigen-specific responses (8) with a distinct immunological profile (9). The induction of high GADA titers was one of the most noticeable outcomes following direct lymph node injection of GAD-alum. This GADA increase was characterized by a shift of subclass distribution, with a marked reduction of the IgG1 fraction in favor of IgG2, IgG3, and IgG4 (7, 9). After 15 months of therapy, the response to GAD_65_ stimulation *in vitro* encompassed enhancement of IL-10 and Th2-cytokine secretion, and loss of cell proliferation. The treatment also induced changes in T cell differentiation and reduced antigen-induced CD8+ T cell activation. Nevertheless, the characterization of the immune response at the cellular level has been restricted so far to T cell populations using a limited number of markers. There is therefore still a need to define cellular responses to intralymphatic delivery of GAD-alum. In the present study we took advantage of mass cytometry to profile major immune cell populations more comprehensively and monitor changes induced after the therapy. Our results show that the *in vivo* effect of the therapy included reduction of naïve and unswitched memory B cells, increase in follicular helper T cells and expansion of Tc2 and PD-1+ CD69+ cells both in CD8+ and in double negative T cells. The treatment also reduced the frequency of double negative NKT and NK cells.

**Figure 1:**
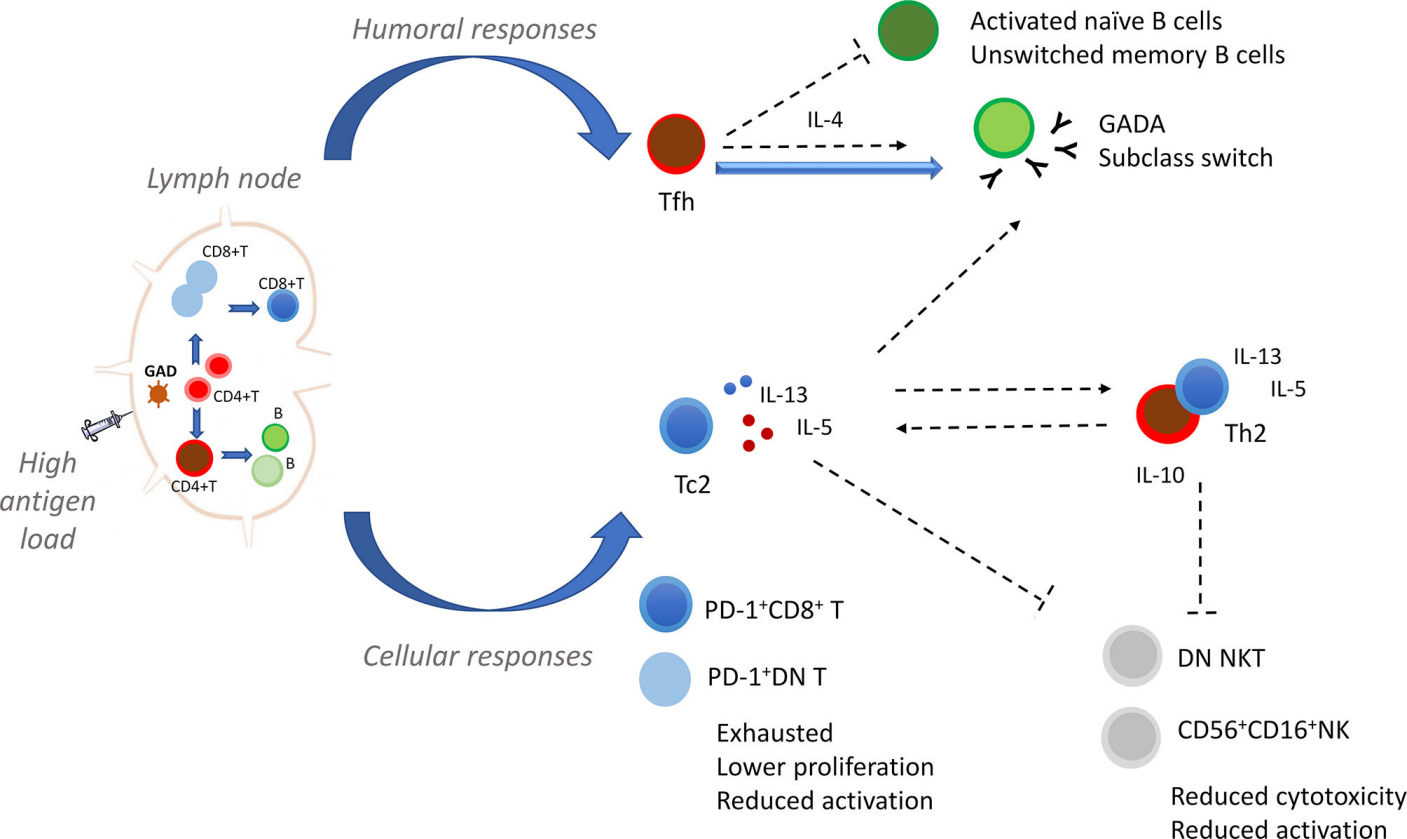
Proposed immunomodulatory effect of GAD-alum injections into the lymph node. Our hypothesis is that the quality of GAD-specific immune responses is determined by direct administration of GAD-alum into the lymph node. Delivery of high antigen dose into the lymph node stimulates antigen-specific T and B cells in GADA positive individuals, and the use of alum, an adjuvant associated with Th2 responses, leads to induction of GAD-specific humoral and cellular immunity, and reinforcement of Th2 immune responses. Immunomodulation of the cellular immune response results in the expansion of CD8+ and double negative (DN) T cells expressing PD-1. We propose that the expansion of these cells may be related to reduced activation of CD8+ T cells and decreased proliferation upon GAD_65_ stimulation. Likewise, the regulation of the humoral immunity given by the feedback loop between follicular helper T cells (Tfh) and B cells is also determined by the high antigen level available for priming CD4+ T cells. Induction of Tfh cells supports their role in the activation and maturation of B cells leading to an increase in GADA titers and shift of GADA subclasses following GAD-alum injections. The Tfh: B interactions may also result in the reduction of activated naïve and unswitched memory B cells. Generation and establishment of a predominant Th2 responses to GAD_65_ might counteract pro-inflammatory factors and generate an environment where Tc2 CD8+ T cells are expanded and further produce IL-5 and IL-13. Restoration of the immunological balance leads to suppression of autoreactive immune responses and reduction of Th1 effector cells, DN NKT and NK cells. Dashed lines indicate possible links.

Findings from the present and previous studies suggest that delivery of high antigen dose directly into the lymph nodes results in the stimulation of antigen-specific T and B cell responses in GADA positive individuals. The use of alum, an adjuvant associated with Th2 responses, leads to the reinforcement of Th2 immune responses that might counteract pro-inflammatory factors and generate an environment where Tc2 CD8+ T cells are expanded. We propose that the expansion of CD8+ and double negative (DN) T cells expressing PD-1 may be related to the reduced activation of CD8+ T cells and decreased proliferation to GAD_65_ (7, 9). Likewise, the regulation of humoral immunity resulting in reduction of naïve and unswitched B cells might be determined by the high level of antigen available for priming follicular helper T cells. Restoration of the immunological balance may lead to suppression of autoreactive immune responses and reduction of Th1 effector cells, double negative NKT and NK cells (**Figure 1**).

## Materials and Methods

### Study Design and Participants

Samples from twelve T1D patients who participated in a single center open-labelled pilot clinical trial (DIAGNODE-1) were included in this study. Individuals aged 12-24 years, received a primary injection of 4 μg each of GAD-Alum (Diamyd Medical, Stockholm) into an inguinal lymph node administrated by the help of ultrasound technique, followed by two booster injections with one-month interval. They also received Vitamin D (Calciferol) in oral solution (2000 U/d) for 4 months, starting 1 month prior to the first GAD-alum injection (7). In the current study, samples from the participants (n=12) collected at baseline and 15 months visits were analyzed.

DIAGNODE trial was approved by the Research Ethics Committee, Linköping University, Sweden (Dnr 2014/153-31) and by the Medical Product Agency, Uppsala, Sweden.

Blood samples were drawn during the morning hours and peripheral blood mononuclear cells (PBMCs) were isolated within 24 h using Leucosep (Greiner Bio One) according to the manufacturer’s instructions and cryopreserved in medium containing 10% DMSO/FCS.

### Cell Stimulation

Cryopreserved PBMCs were thawed and washed with pre-warmed AIM-V medium, and rested overnight at 37°C, 5%CO2. After the resting period, cells were cultured in flat-bottom plates at a density of 4x10^6^ cells/well in AIM-V medium alone with or without 5 μmg/ml rhGAD65 (Diamyd Medical) for 72h at 37°C, 5% CO2.

### Mass Cytometry Staining

The panel of monoclonal antibodies used in this study is described in detail in **Supplementary Table I**. When indicated, purified carrier-free antibodies were conjugated using Maxpar^®^ antibody labelling kit (Fluidigm) according to the manufacturer’s instructions.

PBMCs (4x10^6^) were washed in PBS and stained with 2.5 µM Cell-ID™ Cisplatin (Fluidigm) for 5 min at room temperature to identify dead cells. Cells were then washed in Maxpar^®^ Cell staining buffer (CSB, Fluidigm) and incubated in an extracellular staining cocktail for 30 min at 4°C. PBMC were washed in CSB and resuspended in FOXP3 Fixation/Permeabilization buffer (Thermofisher Scientific) for 40 min. Cells were then washed with Permeabilization buffer (Thermofisher Scientific) and stained with an intracellular staining cocktail for 30 min at 4°C. Samples were then washed in Permeabilization buffer. Finally, cells were stained with 125nM Ir191/193 DNA intercalator (Cell-ID Intercalator-Ir, Fluidigm) and acquired with a CyTOF2^®^ instrument. Data were normalized using EQ Four Element Calibration Beads (Fludigm).

### Data Analysis and Statistics

Data of single, live CD45+ cells from each sample were manually gated (**Supplementary Figure 1A**) and exported using Cytobank platform (https://www.cytobank.org/). Data were then arcsinh-transformed with a cofactor of 5 and a hierarchical stochastic neighbour embedding (HSNE) analysis was performed in Cytosplore+HSNE (10) using default settings. Major cell populations were first identified at the overview level and then, analyzed separately in a semi-supervised data-driven manner (**Supplementary Figure 1B**) as previously described (11). Clustering was performed by Gaussian mean-shift (GMS) in Cytosplore. Clusters with less than 100 cells were excluded from the analysis and clusters showing similar phenotypic characteristics were manually merged. Marker expression levels and cell count in each cluster were exported and statistical analysis was performed in R environment. Packages ComplexHeatmap (v2.8.0) and ggplot2 (v3.3.3) were used to plot data. Wilcoxon signed-rank test was used for pairwise comparisons between visits. P values of <0.05 were considered statistically significant. False discovery rate was controlled using the Benjamini–Hochberg method. A false discovery rate of 15% was set for exploratory purposes.

## Results

**Figure 2:**
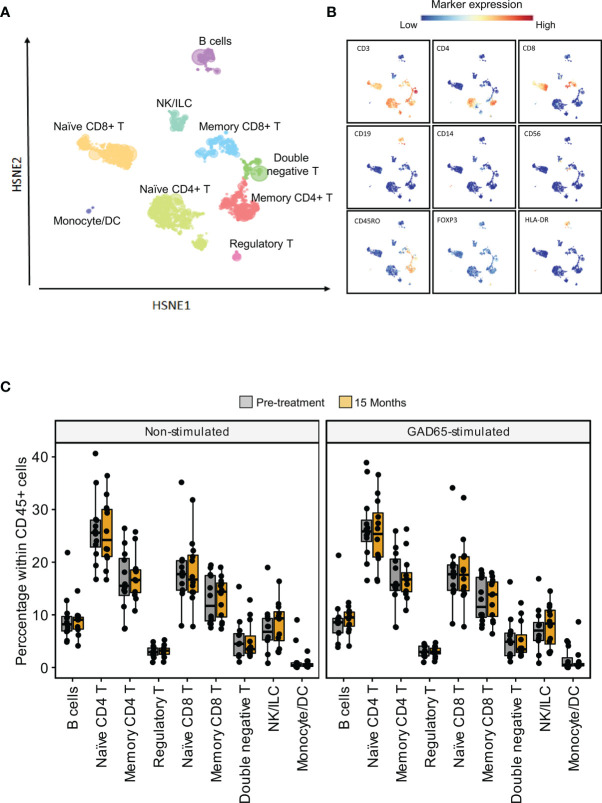
High-dimensional analysis of the main populations within PBMCs **(A)** Data of live CD45+ cells were analyzed using Hierarchical Stochastic Neighbor Embedding (HSNE). Coloured areas depict nine major populations in the HSNE overview level. **(B)** Expression of the markers used the define major populations overlapping the HSNE overview level **(C)** Percentages of the major populations in non-stimulated samples (left) and samples stimulated with GAD_65_ (right) before and after 15 months of treatment.

To profile major immune cell populations, we used a panel of 36 metal-labeled monoclonal antibodies. Surface markers were chosen to identify T and B lymphocytes, natural killer cells, monocytes, and dendritic cells. Antibodies specific for transcription factors, markers of differentiation, activation and function were also included (**Supplementary Table 1**). To reveal the composition of single live CD45+ cells, we applied a hierarchical stochastic neighbor embedding (HSNE) dimensionality reduction analysis and Gaussian mean-shift clustering. Based on the marker expression and density features of the cells, nine distinct major populations were identified at the overview level, namely B cells (CD19+), naïve CD4 T cells (CD3+CD4+CD45RO-), memory CD4 T cells (CD3+CD4+CD45RO+), naïve CD8 T cells (CD3+CD8+CD45RO-), memory CD8 T cells (CD3+CD8+CD45RO+), regulatory T cells (CD3+CD4+CD25+FOXP3+CD127-), double negative T cells (DN T cells, CD3+CD4-CD8-), NK/ILC (CD3-CD19-CD14-CD56+/-) and monocytes and dendritic cells (CD3-CD19-CD14+/- CD11c+/-). The frequency of the main populations did not differ between samples collected before treatment and after 15 months (**Figure 2**).

Next, each major population was selected individually, embedded in lower levels of the HSNE analysis and clustered at the single cell level. Altogether the analysis yielded 103 clusters, defined by a distinctive marker expression profile.

To search for antigen-specific responses induced by the treatment, the response after GAD_65_ stimulation was also analyzed. Clustering analyses of stimulated samples were comparable to those from non-stimulated samples, yielding the same number of clusters and phenotypic characteristics.

Statistical analysis of cell frequency and median marker expression was performed for every cluster. Thereafter, results were focused on cell subsets that differed significantly.

### Unswitched Memory and CXCR5-CD38- Naïve B Cells Were Reduced After GAD-alum Therapy

**Figure 3:**
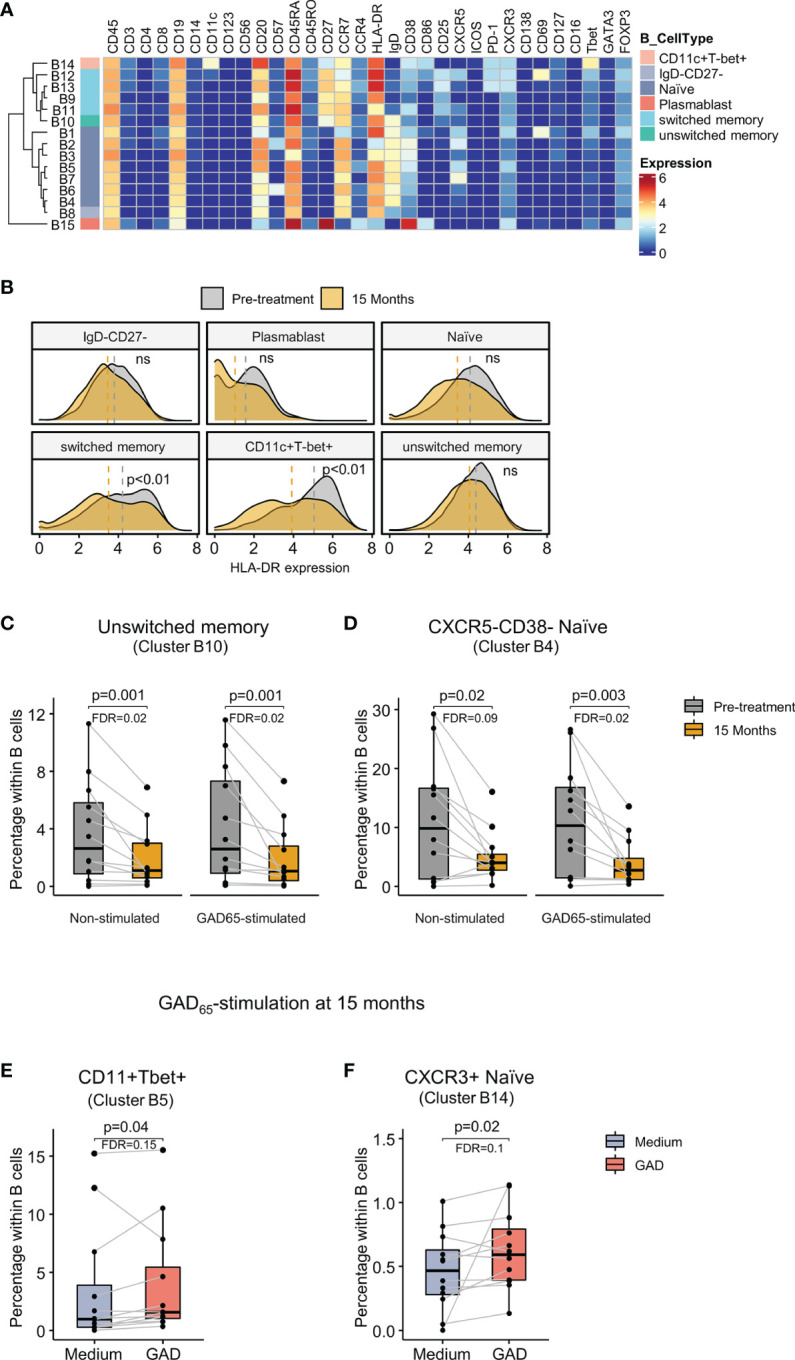
Identification of B cell clusters and changes after GAD-alum therapy. **(A)** Heatmap showing median marker expression across the 15 clusters identified within B cells. **(B)** Histogram depicting the density of HLA-DR expression on naïve, IgD-CD27-, plasmablast, switched memory, unswitched memory and CD11c+Tbet+ B cells before (grey) and after treatment (yellow). Dashed lines indicate median expression. **(C)** Median Proportion of CD27+IgD+ unswitched memory B cells (cluster B10) before (grey) and after treatment (yellow) in non-stimulated and GAD_65_-stimlated samples. **(D)** Proportion of CXCR5-CD38- naïve B cells (cluster B4) before (grey) and after treatment (yellow) in non-stimulated and GAD65-stimulated samples. Proportion of **(E)** CD11c+Tbet+ B cells (cluster B5) and **(F)** CXCR3+ naïve B cells (cluster B14) in 15 months samples cultured in medium alone (blue) or in the presence of 5 μg/ml of GAD_65_ (red) for 72 hours. Dots represent individuals and boxplots indicate median and interquartile range. Wilcoxon signed-rank test. p < 0.05 was considered significant. FDR, False discovery rate (Benjamini–Hochberg). ns: non significant.

Analysis of B cells revealed 15 clusters, including conventional naïve (CD27-IgD+), double negative IgD CD27 (IgD-CD27-), unswitched (CD27+IgD+) and switched (CD27+IgD-) memory cells, plasmablasts (CD27^high^CD38^high^), and a subset of CD11c+Tbet+ (CD11c+Tbet+) B cells (**Figure 3A**). Analysis of marker expression showed a reduction of HLA-DR on switched memory (IgD-CD27+) and CD11c+Tbet+ B cells at 15 months (**Figure 3B**).

Comparison of samples between visits revealed a reduction at 15 months in the proportion of unswitched memory B cells (cluster B10, **Figure 3C**) and CXCR5-CD38- naïve B cells (cluster B4, **Figure 3D**) in both non-stimulated and GAD_65_-stimulated samples.

No differences were observed in the antigen-specific response before treatment. However, analysis of the effect of GAD_65_ recall response showed that GAD stimulation at 15 months increased the percentage of B cells expressing CD11c and T-bet (cluster B5, **Figure 3E**), as well as a subset of CXCR3+ naïve B cells (cluster B14, **Figure 3F**) compared with unstimulated samples from the same visit.

### Follicular Helper T Cells Were Increased While Central Memory Th2-CD4+ T Cells Were Reduced After the Therapy

**Figure 4:**
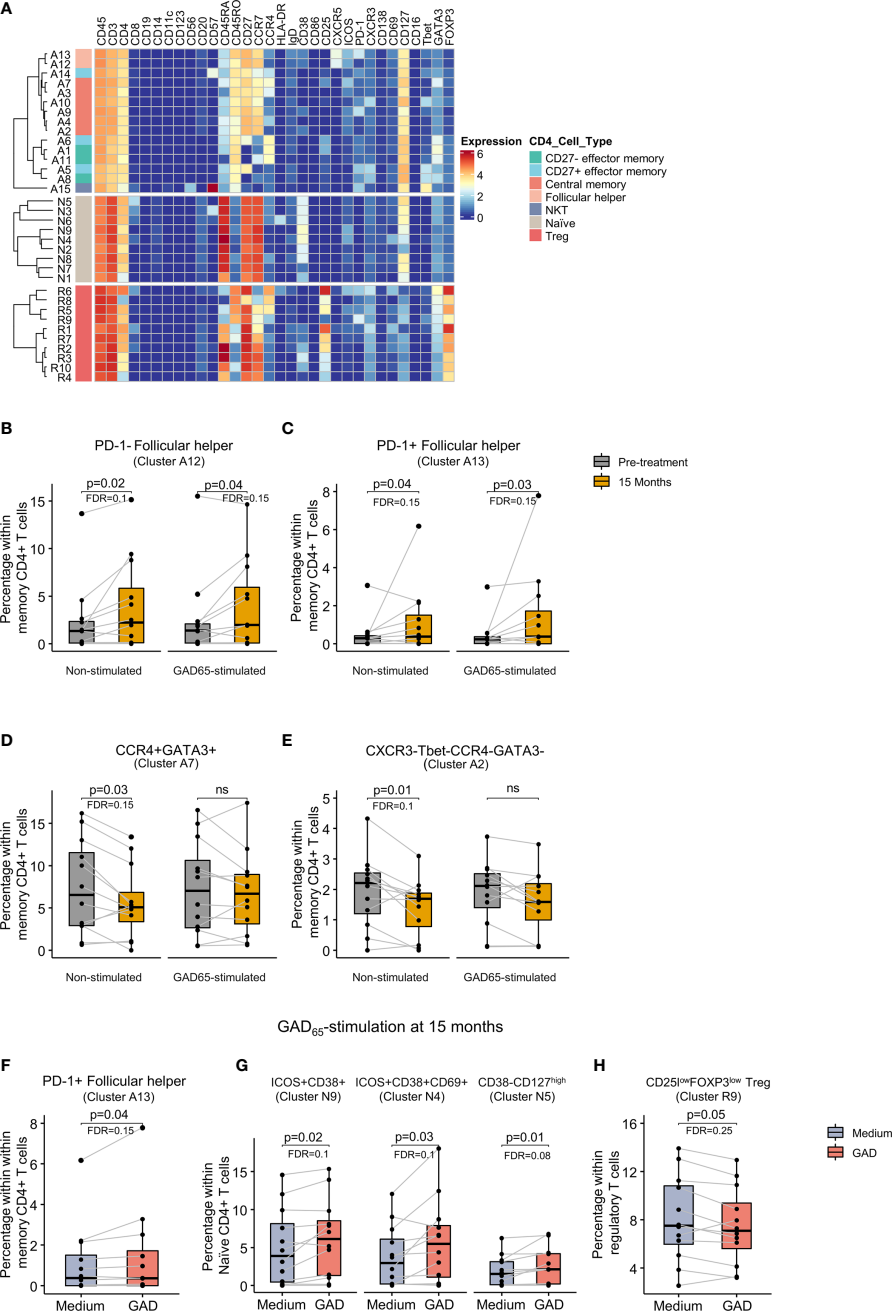
Identification of CD4+ T cell clusters and changes after GAD-alum therapy. **(A)** Heatmap showing median marker expression across the 15 clusters identified within memory CD4+ T cells, the 9 clusters identified within naïve CD4+ T cells and the 10 clusters identified within regulatory T cells. Proportion of **(B)** PD-1- follicular helper T cells (cluster A12), **(C)** PD-1+ follicular helper T cells (cluster A13), **(D)** CCR4+GATA+ (cluster A7) and **(E)** CXCR3- Tbet-CCR4-GATA3- cells (cluster A2) before (grey) and after treatment (yellow) in non-stimulated and GAD_65_-stimulated samples. Proportion after 15 months of treatment of **(F)** PD-1+ follicular helper T cells (cluster A13), **(G)** ICOS+CD38+CD69- (cluster N9), ICOS+CD38 +CD69+ (cluster N4), and CD38-CD127^high^ (cluster N5) naïve CD4+ T cells, and **(H)** CD25^low^FOXP3^low^ within regulatory T cells (cluster R9) in PBMCs cultured in medium alone or in the presence of 5 μcultured in medium alone or in the presence g/ml of GAD_65_ for 72 hours. Dots represent individuals and boxplots indicate median and interquartile range. Wilcoxon signed-rank test. p < 0.05 was considered significant. FDR, False discovery rate (Benjamini–Hochberg). ns: non significant.

Memory CD4+ T cells (15 clusters) were stratified based on the expression of CCR7, CD27 and CD45RA into central memory (CCR7+CD27+CD45RA-), CD27+ effector memory (CCR7-CD27+CD45RA-), and CD27- effector memory (CCR7-CD27-CD45RA-). Within these subpopulations, the expression of chemokine receptors and transcription factors further defined Th1 (CXCR3+Tbet+) and Th2 (CCR4+GATA3) cells. Additional heterogeneity was introduced by the expression of activation and inhibitory markers. Subsets of follicular helper T cells (Tfh, CXCR5+) and CD4+ natural killer T cells (CD56+) were also identified (**Figure 4A**).

Analysis of memory CD4+ T cells, revealed two distinct clusters of Tfh cells differentiated by the expression of PD-1. Comparison between visits showed that these two clusters, PD-1- (cluster A12) and PD-1+ (cluster A13), were more abundant after 15 months in both non-stimulated and GAD_65_-stimulated samples (**Figures 4B, C**). In contrast, two central memory subsets, GATA3+CCR4+ Th2 cells (cluster A7) and a subset that did not display Th2 or Th1 markers (cluster A2), were reduced after 15 months in non-stimulated samples (**Figures 4D, E**). A similar trend was observed after GAD_65_ stimulation without being statistically significant.

Analysis of the antigen-specific response did not reveal differences before treatment. However, stimulation with GAD_65_ at 15 months increased the frequency of the PD-1+ Tfh cells compared to samples from the same visit cultured in medium alone (**Figure 4F**). Other changes induced by antigen recall were also detected in naïve CD4+ T cells and included the detection of higher frequencies of two subsets of ICOS+CD38^high^ cells differentiated by the expression of CD69 (clusters N9 and N4, respectively), and another cluster of naïve CD38-CD127^high^ cells (cluster N5) (**Figure 4G**). Within regulatory T cells, stimulation with GAD65 reduced the frequency of a cluster of PD-1+ cells expressing low levels of CD25 and FOXP3 (cluster R9) as compared to cells cultured in medium alone (**Figure 4H**).

### PD-1-Expressing Memory CD8+ T and Tc2 Cells Were Increased After GAD-alum Therapy

**Figure 5:**
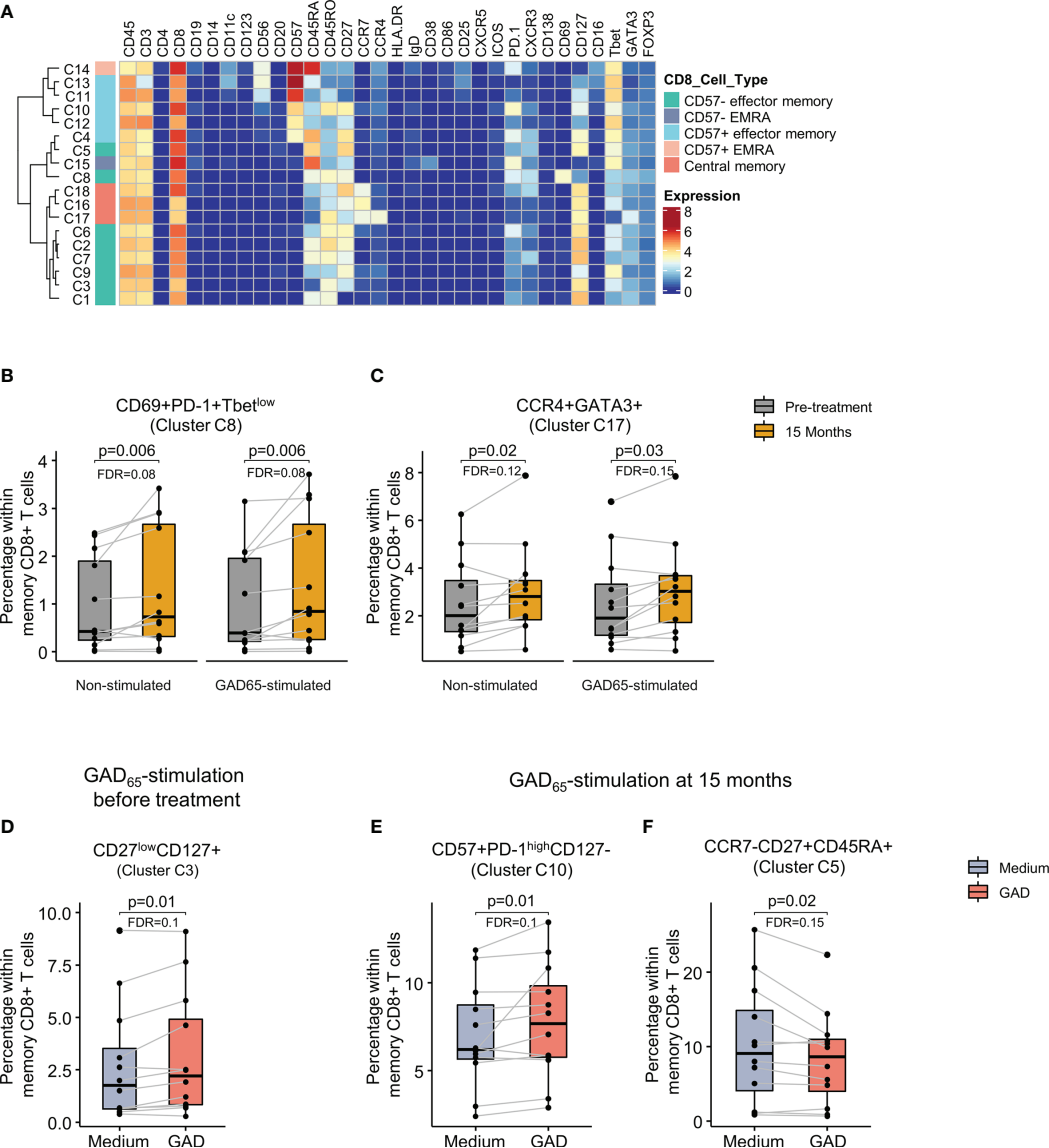
Identification of memory CD8+ T cell clusters and changes after GAD-alum therapy. **(A)** Heatmap showing median marker expression across the 18 clusters identified within memory CD8+ T cells. Proportion of **(B)** CD69+PD-1+Tbet^low^ cells (cluster C8) and **(C)** CCR4+GATA3+ cells (cluster C17) within memory CD8+ T cells before (grey) and after treatment (yellow) in non-stimulated and GAD65-stimulated samples. **(D)** Proportion of CD27^low^CD127+ memory CD8 + T cells (cluster C3) in PBMCs collected before treatment and cultured in medium alone or in the presence of 5 μg/ml of GAD_65_ for 72 hours, and proportion of **(E)** CD57+PD-1^high^CD127- (cluster C10) and **(F)** CCR7-CD27+CD45RA+ (cluster C5) memory CD8+ T cells in PBMCs collected after 15 months of treatment and cultured in medium alone or in the presence of 5 μg/ml of GAD_65_ for 72 hours. Dots represent individuals and boxplots indicate median and interquartile range. Wilcoxon signed-rank test. p < 0.05 was considered significant. FDR, False discovery rate (Benjamini–Hochberg).

Memory CD8+ T cells (18 clusters) were also defined by the expression of CCR7, CD27 and CD45RA, and further heterogeneity was introduced by the expression of CD57, T-bet and different expression levels of activation and inhibitory molecules and chemokine receptors (**Figure 5A**).

Comparison between visits revealed that effector cells expressing PD-1, CD69, CXCR3 and low levels of T-bet (cluster C8) were more abundant after 15 months than before the treatment both in non-stimulated and in GAD_65_-stimulated samples (**Figure 5B**). In addition, a cluster of central memory CD8+ T cells displaying a Tc2 phenotype (CCR4+GATA3+, cluster C17) was also significantly increased after in the same samples (**Figure 5C**).

Analysis of the antigen-specific response following stimulation with GAD_65_ revealed an increase in CD27-CD127+ effector memory cells (cluster C3) in samples obtained before the treatment (**Figure 5D**). In contrast, GAD_65_- stimulation of 15 months samples induced CD27+CD57+ PD-1^high^ memory CD8+ T cells (Cluster C10, **Figure 5E**) and reduced the proportion of CCR7- CD27+ CD45RA+ effector cells (cluster C5, **Figure 5F**) as compared to samples cultured in medium alone.

### Double-Negative NKT and CD56+CD16+ NK Cells Were Reduced While PD-1+CD69+ Double-Negative T Cells Increased After the Therapy

**Figure 6:**
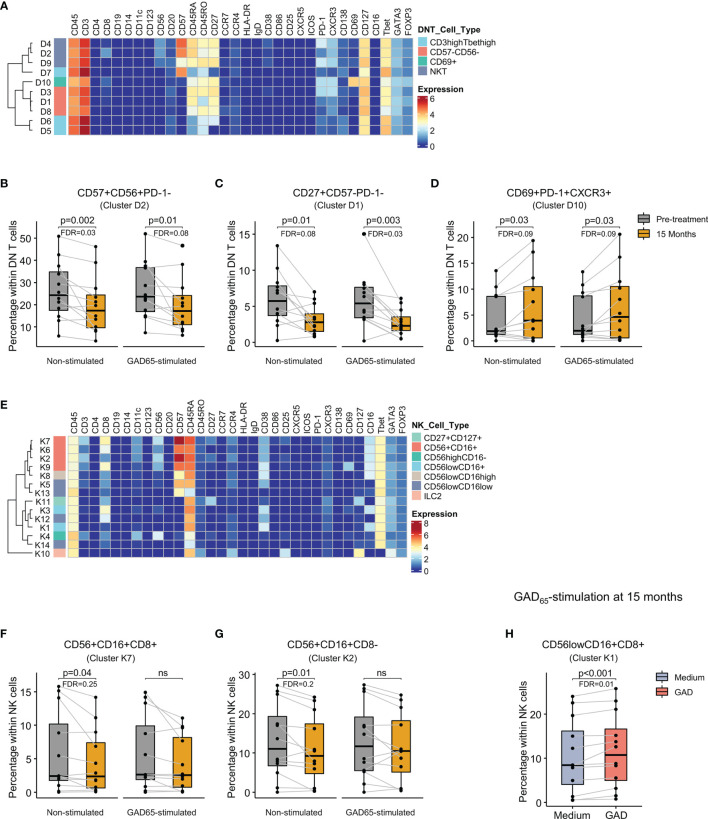
Identification of cell clusters within double negative T and NK cells and changes after GAD-alum therapy. **(A)** Heatmap showing median marker expression across the 10 clusters identified within double negative T cells. Proportion of **(B)** CD57+CD56+PD-1- (cluster D2), **(C)** CD27+CD57-PD-1-(cluster D1), and **(D)** CD69+PD-1+CXCR3+ (cluster D10) double negative T cells before (grey) and after treatment (yellow) in non-stimulated and GAD65-stimulated samples. **(E)** Heatmap showing marker expression across the 14 clusters identified within NK cells. Proportion of **(F)** CD56+CD16+CD8+ (cluster K7), **(G)** CD56+CD16+CD8- (cluster K2) NK cells before (grey) and after treatment (yellow) in non-stimulated and GAD_65_-stimulated samples. **(H)** Proportion after 15 months of treatment of CD56^low^CD16+CD8+ NK cells in PBMCs cultured in medium alone (blue) or in the presence of 5 μg/ml of GAD_65_ (red) for 72 hours. Dots represent individuals and boxplots indicate median and interquartile range. Wilcoxon signed-rank test. p < 0.05 was considered significant. FDR, False discovery rate (Benjamini–Hochberg); ns, non significant.

Analysis of DN T cells identified 10 clusters with memory effector phenotype distributed in 3 major groups: CD3^high^Tbet^high^ cells, double negative natural killer T cells (CD56+CD57+) and CD56-CD57- cells (**Figure 6A**). Comparison of cluster distribution between baseline and 15 months revealed a reduction of CD56+CD57+PD-1- (cluster D2) and CD27+CD57-PD-1- (cluster D1) cells at 15 months both in non-stimulated and in GAD_65_-stimulated samples (**Figures 6B, C**). In contrast, a subset of CD56-CD57- cells expressing CD69, PD-1 and CXCR3 (cluster D10) increased after 15 months (**Figure 6D**).

Among NK/ILC population, 14 clusters were identified, including conventional CD56^high^CD16-, CD56+CD16+, CD56^low^CD16^high^, and CD56^low^CD16^low^ subsets, group 2 innate lymphoid cells (ILC2, CD127+CCR4+CD25+GATA3+), and other clusters expressing CD127 and CD27 (**Figure 6E**). Differences following the treatment included lower frequency of CD56+CD16+ CD57+cells with or without CD8 expression (cluster K7 and K2, respectively) in non-stimulated samples after 15 months (**Figures 6F, G**). GAD_65_ stimulation at 15 months increased the proportion of CD56^low^CD16+CD8+ cells expressing high levels of CD38 (cluster K1) compared to samples cultured in medium alone (**Figure 6H**).

### Slower C-Peptide Loss After 15 Months Was Associated With Reduction of Double-Negative NKT Cells

**Figure 7:**
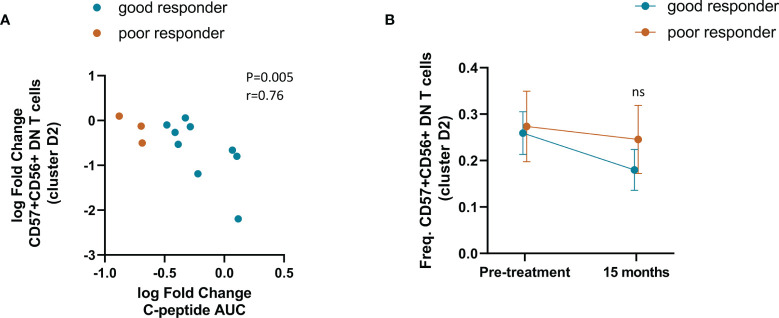
Association between double negative NKT cells with C-peptide preservation after 15 months. **(A)** Correlation between the change in the frequency of CD57+CD56+ DN T cells and the change in C-peptide from before treatment to 15 months. Spearman correlation was performed. **(B)** Frequency over time of CD57+CD56+ DN T cells in good responders (n=9) and poor responders (n=3). Mann-Whitney test was performed to compare the difference between the groups. ns, no significative.

We investigated if any of the observed changes were associated with C-peptide preservation after 15 months. Change in the frequency of CD57+CD56+ DN T cells (cluster D2) inversely correlated with the fold change of C-peptide AUC (**Figure 7A**). Stratification of patients into good and poor responders showed a more pronounced decrease in the frequency of CD57+CD56+ DN T cells in individuals with better response although non statistically significant (**Figure 7B**). No other association between clinical response and cell subsets was found.

## Discussion

In this study, we sought to gain insight into immune cell responses following intralymphatic injections of GAD-alum. Using a mass cytometry panel designed to provide an in-depth profile of the immune landscape, we were able to unveil immunological changes in a relatively small group of participants in an open label clinical trial.

Among the immunomodulatory effects induced by the treatment, we observed a reduction of naïve B cells lacking CXCR5 and CD38, a phenotype attributed to activated cells. Loss of anergy (12) and defects in naïve B cell function leading to expansion of autoreactive cells (13–15) have been reported in T1D patients. This raises the question of whether GAD-alum therapy reduced autoreactive naïve B cells that had been expanded before the treatment. If so, it is possible that the microenvironment generated by GAD administration contributed to the reestablishment of naïve B cell function. Alternatively, the decline in activated naïve B cells may be due to dynamic changes during the disease. One interesting finding was the marked reduction of unswitched memory B cells. Although their development and function are not well understood, it has been suggested that they represent circulating marginal zone B cells that exert their function in T cell-independent immune responses (16). However, other studies have reported that they originate in germinal center (GC) reactions and, unlike switched memory cells, preferably re-enter GC upon re-encounter with antigen (17). Thus, it might be reasonable that the recruitment of unswitched memory cells into GC after GAD-alum injections led to a reduction of their circulating fraction.

Stimulation with GAD_65_ resulted in the expansion of B cell subsets with activated phenotype at 15 months. This was not observed in samples obtained prior to treatment, suggesting an effect linked to the therapy. Of note, GAD_65_ stimulation induced B cells expressing T-bet and CD11c. These markers define activated effector B cells that are expanded by IL-21 produced by Tfh cells and are poised to differentiate into antibody secreting cells (18, 19). We have previously shown that GAD-alum injections enhanced the levels of GADA with a shift of subclass distribution (7). Thus, induction of Tbet+CD11c+ B cells suggests a role of these cells in the increment of GADA after treatment. Another intriguing finding was the reduction of HLA-DR expression on memory B cells, suggesting reduced antigen presentation capacity of these cells following therapy.

One particularly interesting finding was the observed expansion of circulating Tfh cells following GAD-alum treatment, consistent with the idea that activation of B cells leading to autoantibody production plays a role in the immunomodulatory effect of the therapy. A link between Tfh cells and generation of autoantibodies has been suggested in a few studies reporting increased frequencies of circulating Tfh cells in established T1D, newly diagnosed T1D, and multiple autoantibody-positive children close to diagnosis (20, 21). Tfh cells are known to be specialized in providing the support that B cells need for maturation, class switching and production of antibodies in germinal centers (22). Although this function takes place in the follicles, it is thought that the abundance of circulating Tfh cells reflects their activity in the lymph nodes (23). Since Tfh cell responses are sustained by high load and continuous antigen stimulation in the follicles (24), direct injection of GAD-alum into the lymph node might increase the level of antigen fostering GC reactions. Interestingly, stimulation with GAD_65_ at 15 months increased the fraction of activated PD-1+ Tfh cells, considered the most efficient at promoting B cell differentiation into antibody secreting cells upon antigen re-exposure (23). Altogether, our findings suggest that the dramatic increase in GADA after intralymphatic GAD-alum administration (7) might be mediated by Tfh cells induced by the treatment.

We have previously shown that a reduction of the T-cell central memory pool in favor of an effector phenotype ocurred in the same patients after 15 months (7). Here we extend our findings and show that reduction of central memory CD4+ T cells was driven by Th2 cells (expressing CCR4 and GATA3) and by cells that did not express markers associated with Th1 (T-bet, CXCR3) or Th2 (GATA3, CCR4) differentiation. Although no change in effector cells with Th2 phenotype was observed, we cannot rule out the possibility that the scarce number of GAD-specific Th2 cells generated in response to GAD-alum precluded their identification in our analysis. Another interesting observation was the subtle expansion of CCR4+GATA3+CD8+ T cells (Tc2), known as a source of IL-5 and IL-13 (25). Like CD4+ T cells, CD8+ T cells exhibit plasticity and can redirect their functional program depending on the microenviromoment. For instance, IFNγ-producing CD8+ T-cells exposed to IL-4 upregulate GATA3 and produce IL-13 upon antigen stimulation (26). Indeed, we have previously reported that IL-5 and IL-13 are secreted by PBMC as part of the response to *in vitro* stimulation with GAD_65_ in the same group of patients (7) Thus, the cytokine milieu induced by the treatment might explain the increase in Tc2 cells. Whether these cells further contribute to the production of IL-5 and IL-13 should be worth future research.

Besides markers essential for the identification of immune cell populations, our panel included antibodies against targets used to assess functional state. One of the most striking findings in this study was the expansion of CD8+ T cell subsets expressing PD-1, an inhibitory marker commonly present in exhausted cells (27). T cell exhaustion is a distinct differentiation state characterized by reduced proliferation, cytotoxicity, and cytokine release caused by high antigen load and persistent stimulation (28). While exhausted CD8+ T cells (Tex) limit the immune response in chronic viral infections and cancer, they have also been associated with favorable outcome in autoimmunity (29). Expansion of different Tex subsets has been linked with response to T cell-targeting therapies in T1D (30, 31). The PD-1+ CD8+ T cell subset detected by us resembles a recently described tissue-resident terminal Tex (32) in that they express CD69, low levels of Tbet, and lack CXCR5. Moreover, they exhibit CXCR3, a feature of islet-specific Tex that has been associated with slow disease progression in established T1D (33). Interestingly, a subset of DN T cells displaying similar phenotype (CD69+, PD-1+) was also expanded after GAD-alum treatment. DN T cells can derive from CD8+ T cells that lose CD8 expression upon antigen encounter (34), and it has been suggested that self-reactive DN T cells upregulate PD-1 and Helios in a process similar to exhaustion (35). Our observation that GAD_65_ recall also induced an exhausted-like CD8+ T cell subset expressing high levels of PD-1 and CD57, suggests that both PD-1+CD8+ and PD-1+DN T cells could have been generated from a pre-existing pool of GAD-specific CD8+ T cells.

Although one of the postulated effects of antigen-specific immunotherapy is the induction of Tregs able to maintain peripheral tolerance (1), deep characterization of Tregs did not reveal any relevant effect of GAD-alum therapy in this population. This is line with our previous results using a more limited panel of T cell markers (8, 36). Differences induced on the Treg compartment by GAD_65_ stimulation were limited to CD45RA+ cells expressing low levels of CD25 and FOXP3, representing the non-suppressive fraction of Treg (7, 8). Other variations observed during the treatment included the reduction of cells commonly associated with cytotoxic activity such as DN NKT cells and CD56+CD16+ NK cell subsets. However, it is difficult to assure whether the decline was a result of the treatment, as variations in cytotoxic NK cell subsets might also be related to dynamic changes during the disease (37).

Differences observed after antigen stimulation support the notion that they are a result of the immunomodulatory effect induced by GAD-alum injections. Furthermore, most changes induced by *in vitro* culture with GAD_65_ were solely seen in samples after 15 months and not in those collected prior to treatment. Of note, results observed after GAD_65_-recall at 15 months are functionally linked to other changes observed throughout the study. For instance, T-B cell interactions leading to GADA production (7) are highlighted by the antigen-specific induction of activated PD-1+ Tfh cells along with B cells expressing T-bet and CD11c. Likewise, recall response favored CD8+ T cells with inhibitory or “exhausted-like” phenotype over effector cells with a phenotype commonly associated with cytotoxic activity, in line with our previous findings showing decreased activation and proliferation (7). Nevertheless, since the pilot trial did not include a placebo group, it is uncertain whether changes observed in unstimulated cell cultures were due to a direct effect of treatment or the course of the disease, and the results should be validated in larger placebo-controlled trials.

Although the low number of individuals ruled out reliable statistical comparisons with clinical responses, it was interesting that the reduction of DN NKT cells was linked to better clinical outcomes in patients. However, we should be very careful in drawing any conclusion from this finding, as there is little indication that the magnitude of changes in immune populations following antigen administration reflects the extension of metabolic outcomes. After all, such correlates have not even been found for immunogenic vaccines (38).

In summary, our data suggest that direct administration of GAD-alum into the lymph nodes leads to modulation of Tfh and B cell responses, induces Tc2 and CD8+ T cells with an exhausted-like phenotype and reduces the proportion of potentially cytotoxic CD56+ DN T cells. Although activation of regulatory mechanisms capable of suppressing harmful autoreactive responses is a desired effect of antigen-specific immunotherapies, our results suggest that the therapy does not necessarily induce tolerance or unresponsiveness but rather modulates the quality of the existing immune response to the antigen. It might be possible that while GAD-specific humoral immune response is enhanced, cellular immunity provided by CD8+T cells becomes exhausted, resulting in the reduction of deleterious effector functions.

## Data Availability Statement

The raw data supporting the conclusions of this article will be made available by the authors, without undue reservation.

## Ethics Statement

The studies involving human participants were reviewed and approved by Research Ethics Committee, Linköping University, Sweden (Dnr 2014/153-31). Written informed consent to participate in this study was provided by the participants’ legal guardian/next of kin.

## Author Contributions

HB and RC designed the experiments. HB performed experiments. HB and MP developed and optimized mass cytometry panel. MP conjugated antibodies and acquired mass cytometry data. HB analyzed the data. HB and RC interpreted the results and wrote the manuscript. JW recruited and followed patients. JL reviewed the manuscript and conceived DIAGNODE-1 study. All authors read and approved the final manuscript.

## Funding

This work was supported by Barndiabetesfonden (Swedish Child Diabetes Foundation), Swedish Diabetes research foundation, and an unrestricted grant from Diamyd Medical. The funders were not involved in the study design, collection, analysis, interpretation of data, the writing of this article or the decision to submit it for publication.

## Conflict of Interest

The authors declare that the research was conducted in the absence of any commercial or financial relationships that could be construed as a potential conflict of interest.

## Publisher’s Note

All claims expressed in this article are solely those of the authors and do not necessarily represent those of their affiliated organizations, or those of the publisher, the editors and the reviewers. Any product that may be evaluated in this article, or claim that may be made by its manufacturer, is not guaranteed or endorsed by the publisher.
