# Supplementary material for: Intralymphatic GAD-alum Injection Modulates B Cell Response and Induces Follicular Helper T Cells and PD-1+ CD8+ T Cells in Patients With Recent-Onset Type 1 Diabetes
Source: Front Immunol. 2022 Jan 12;12:797172. doi: 10.3389/fimmu.2021.797172 (PMC8791064; doi:10.3389/fimmu.2021.797172)
Supplement: Supplementary file 1 [file DataSheet_1.pdf]

Supplementary Material

1 Supplementary Table 1

2 Supplementary Figure 1

Supplementary Table 1. Staining panel and details of antibodies. * Conjugated in-house. EC: extracellular cocktail. IC: intracellular cocktail

Metal tag	Target	Clone	Supplier	Usage
**89Y**	CD45	HI30	Fluidigm	EC
**141 Pr**	CCR6	GO34E3	Fluidigm	EC
**142 Nd**	CD19	HIB19	Fluidigm	EC
**143 Nd**	ICOS	C398.4A	Fluidigm	EC
**145 Nd**	CD4	RPA-T4	Fluidigm	EC
**146 Nd**	IgD	IA6-2	Fluidigm	EC
**147 Sm**	CD11c	Bu15	Fluidigm	EC
**148 Nd**	CD16	3G8	Fluidigm	EC
**149 Sm**	CD25	2A3	Fluidigm	EC
**150 Nd**	CD8a	RPA-T8	Biolegend*	EC
**151 Eu**	CD123	6H6	Fluidigm	EC
**152 Sm**	CD56	HCD56	Biolegend*	EC
**153 Eu**	CCR4	205410	Fluidigm	EC
**154 Sm**	CD57	HNK-1	Biolegend*	EC
**155 Gd**	CD27	L128	Fluidigm	EC
**156 Gd**	CD86	IT2.2	Fluidigm	EC
**159 Tb**	CCR7	GO43H7	Fluidigm	EC
**160 Gd**	Tbet	4B10	Fluidigm	IC
**161 Dy**	CD69	FN50	Biolegend*	EC
**162 Dy**	FOXP3	PCH101	Fluidigm	IC
**163 Dy**	CXCR3	G025H7	Fluidigm	EC
**164 Dy**	CXCR5	RF8B2	Fluidigm	EC
**165 Ho**	CD45RO	UCHL1	Fluidigm	EC
**166 Er**	PD-1	EH12.2H7	Fluidigm*	EC
**167 Er**	GATA3	TWAJ	Fluidigm	IC
**168 Er**	CD138	DL-101	Fluidigm	EC
**169 Tm**	CD45RA	HI100	Fluidigm	EC
**170 Er**	CD3	UCHT1	Fluidigm	EC
**171 Yb**	CD20	2H7	Fluidigm	EC
**172 Yb**	CD38	HIT2	Fluidigm	EC
**174 Yb**	HLA-DR	L243	Fluidigm	EC
**175 Lu**	CD14	M5E2	Fluidigm	EC
**176 Yb**	CD127	A019D5	Fluidigm	EC
**191/193 Ir**	DNA		Fluidigm	DNA
**195 Pt**	Cisplatin		Fluidigm	Live/Dead

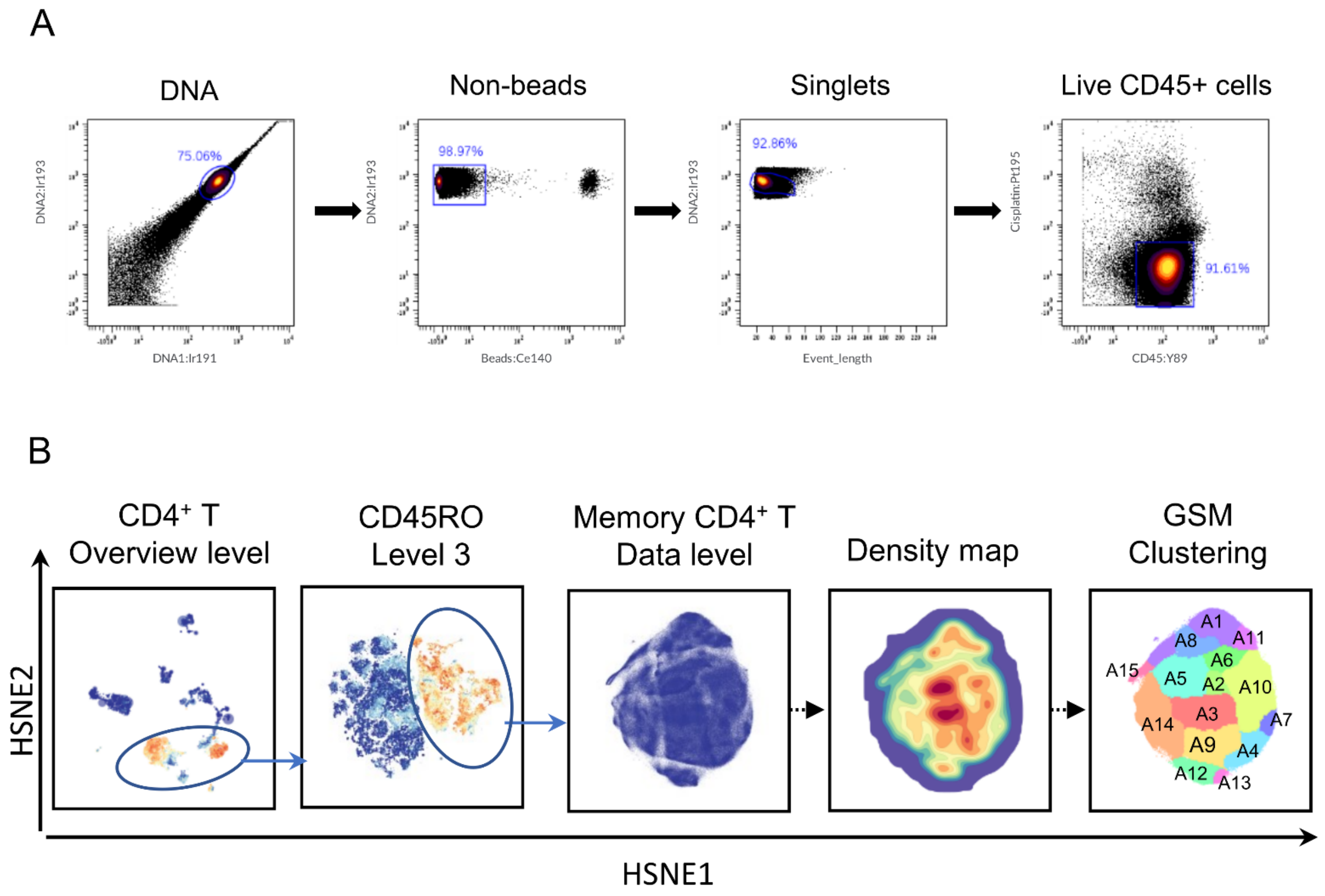

Supplementary Figure 1. Data cleaning and hierarchical analysis strategy. (A) Gating strategy for live single CD45$^+$ cells. (B) Example of the hierarchal data exploration applied to each major population. The CD4+ T cell population was selected at the overview level. Memory CD4+ T cells were selected at level 3 based on CD45RO expression. At the data level, unsupervised Gaussian mean-shift (GMS) clustering used the local probability density of HSNE-embedded cells (density map) to identify phenotypically distinct clusters.
